# People with chronic ankle instability benefit from brace application in highly dynamic change of direction movements

**DOI:** 10.1186/s13047-021-00452-0

**Published:** 2021-02-17

**Authors:** Patrick Fuerst, Albert Gollhofer, Markus Wenning, Dominic Gehring

**Affiliations:** 1 Department of Sport Science, University of Freiburg, Sandfangweg 4, 79102 Freiburg i. Br, Germany; 2 Department of Orthopedics and Trauma Surgery, Medical Faculty, University Medical Center, University of Freiburg, Hugstetter Str. 55, 79106 Freiburg, Germany

**Keywords:** Chronic ankle instability, Ankle brace, Ankle injuries, Injury prevention, Cutting, Kinematics, Biomechanics

## Abstract

**Background:**

The application of ankle braces is an effective method for the prevention of recurrent ankle sprains. It has been proposed that the reduction of injury rates is based on the mechanical stiffness of the brace and on beneficial effects on proprioception and neuromuscular activation. Yet, how the neuromuscular system responds to the application of various types of ankle braces during highly dynamic injury-relevant movements is not well understood. Enhanced stability of the ankle joint seems especially important for people with chronic ankle instability. We therefore aimed to analyse the effects of a soft and a semi-rigid ankle brace on the execution of highly dynamic 180° turning movements in participants with and without chronic ankle instability.

**Methods:**

Fifteen participants with functional ankle instability, 15 participants with functional and mechanical ankle instability and 15 healthy controls performed 180° turning movements in reaction to light signals in a cross-sectional descriptive laboratory study. Ankle joint kinematics and kinetics as well as neuromuscular activation of muscles surrounding the ankle joint were determined. Two-way repeated measures analyses of variance and post-hoc t-tests were calculated.

**Results:**

Maximum ankle inversion angles and velocities were significantly reduced with the semi-rigid brace in comparison to the conditions without a brace and with the soft brace (*p* ≤ 0.006, *d* ≥ 0.303). Effect sizes of these reductions were larger in participants with chronic ankle instability than in healthy controls. Furthermore, peroneal activation levels decreased significantly with the semi-rigid brace in the 100 ms before and after ground contact. No statistically significant brace by group effects were found.

**Conclusions:**

Based on these findings, we argue that people with ankle instability in particular seem to benefit from a semi-rigid ankle brace, which allows them to keep ankle inversion angles in a range that is comparable to values of healthy people. Lower ankle inversion angles and velocities with a semi-rigid brace may explain reduced injury incidences with brace application. The lack of effect of the soft brace indicates that the primary mechanism behind the reduction of inversion angles and velocities is the mechanical resistance of the brace in the frontal plane.

## Background

The ankle joint is one of the most frequently injured sites of the human body [1, 2]. As ankle injuries are responsible for a considerable amount of time loss and can have long-lasting consequences for athletes [3], a lot of effort has been put into the development of different prevention strategies [4–7].

The application of ankle braces is a successful preventive approach, which has the potential to reduce ankle injuries by 50–70 % [4, 8–10]. It has been proposed that the primary mechanism behind the reduction of injuries is that the brace provides additional mechanical stiffness to the ankle joint [11]. This may lead to a more neutral joint position already at foot strike and may restrict excessive inversion angles during ground contact to a healthy range [12, 13]. Additionally, braces may reduce inversion velocities and secure more time for the surrounding muscles to counteract the movement and to stabilize the ankle joint actively [14, 15]. Experimentally, ankle braces have already been shown to reduce ankle inversion angles and velocities during landings and during simulated inversion movements on tilt platforms [16–20], in which semi-rigid braces were able to produce larger reductions than soft braces [17, 19, 20]. The effects of ankle bracing on ankle joint kinematics during injury-relevant run-and-cut movements, however, are not well understood and the need for studies in this field has been expressed [21]. A question which needs to be addressed is whether the mechanical support is able to significantly stabilize the ankle joint against the enormous forces that appear during these dynamic movements in competitive sports situations.

In addition to these mechanical effects, improved neuromuscular function has been discussed as another contributing factor to the preventive effect of ankle braces [4, 22, 23]. Increased neuromuscular activation through enhanced proprioceptive feedback could provide higher joint stiffness and active joint stabilization [24–26]. Yet, contrary to this hypothesis, a number of studies have shown that ankle braces instead tend to reduce peroneal activation in walking, jogging, running, on tilt platforms and during functional exercises [16, 27–29]. How the neuromuscular system responds to ankle bracing in more dynamic, injury-relevant tasks like cutting or turning, however, has yet to be determined [28].

Enhanced stabilization of the ankle joint seems especially important for people who suffer from chronic ankle instability (CAI), whose ankle joint control is impaired. CAI is a complex phenomenon which is thought to be caused by various impairments, which can be used to define different subtypes of CAI: mechanical instability (MI), which refers to structural changes of the ankle joint, and functional instability (FI), which is typically associated with impairments in neuromuscular control of the ankle joint [30, 31]. Interestingly, these two dimensions of insufficiencies are in analogy to the above-mentioned modes of operation of ankle braces, i.e., providing mechanical stability and improving neuromuscular function. As a consequence, people suffering from different subtypes of CAI may benefit from the application of ankle braces through different pathways: braces may counteract neuromuscular deficits in people with FI, whereas people with MI may benefit more from the mechanical resistance of an ankle brace.

Therefore, the aim of the present study was to evaluate the effects of two types of braces with different levels of mechanical stiffness, i.e., a soft brace (MalleoTrain® S open heel, Bauerfeind AG, Zeulenroda, Germany) and a semi-rigid brace (MalleoLoc®, Bauerfeind AG, Zeulenroda, Germany), on the execution of highly dynamic movements with a 180° change of direction in participants with different subtypes of CAI. Ankle joint kinematics and kinetics as well as neuromuscular activation of muscles surrounding the ankle joint were analysed in participants with isolated FI, in participants with a combination of both FI and MI and in a control group with healthy ankle joints. We hypothesized (a) that both braces would reduce ankle inversion angles, velocities and moments and provoke decreased peroneal muscle activation in all participants, (b) that those effects would be larger in participants with CAI and (c) that the semi-rigid brace would show more pronounced effects in participants with a combination of FI and MI than in participants with FI, because its mode of operation may be most suitable to counteract mechanical insufficiencies.

## Methods

### Participants

Three groups of physically active participants were included in a cross-sectional descriptive laboratory study: A healthy control group with 15 participants (CON) and two groups of participants with CAI, which differed in their mechanical ankle stability status. Fifteen CAI participants formed a group with isolated functional ankle instability (FI) and 15 participants composed a group with both functionally and mechanically unstable ankle joints (FMI; see Table 1).

Functional instability was tested with the Cumberland Ankle Instability Tool (CAIT) [32, 33]. Mechanical instability was determined for all participants in a manual examination by the same orthopaedic surgeon, who was blinded to CAIT scores and ankle injury history. The mechanical stability status of the tested ankles was described on a three-point rating scale from 0 (physiological anterior talar drawer/talar tilt – mechanically stable) to 2 (pathological anterior talar drawer/talar tilt – mechanically unstable) [33].

Participants in the control group had CAIT scores of 30 points, were rated as mechanically stable, and had no history of ankle injuries. Participants in the FI- and FMI-group had CAIT scores of ≤ 24 points [33, 34] and an injury history of two or more ankle sprains at the tested ankle with the last sprain sustained within two years prior to data collection (Table 1) [35]. They only differed in their mechanical stability status: the FI-group had mechanically stable ankles, whereas the FMI-group had mechanically unstable ankle joints. Participants with a mechanical stability rating of “1”, participants with acute leg injuries or with a history of fractures or surgery at the tested ankle were excluded from the study [35].

Participants from all groups were matched for age, gender, height and body weight. Participants in the FI- and FMI-group were further matched pairwise for CAIT score of the tested ankle (Table 1).

**Table 1:** Characteristics of Participants (Mean ± SD)

Group	Gender	Age, years	Height, cm	Body Weight, kg	CAIT-Score	Number of ankle sprains		Time since last ankle sprain, months	
CON	5 ♂ 10 ♀	23.0 ± 2.7	174.5 ± 9.2	69.1 ± 10.1	30.0 ± 0.0	0:	n = 18	-	
FI	6 ♂ 9 ♀	23.1 ± 2.7	175.7 ± 9.1	71.8 ± 14.0	19.7 ± 3.5	2–3:	n = 8	< 6:	n = 8
						3–5:	n = 3	6–12:	n = 3
						> 5:	n = 4	12–24:	n = 4
FMI	5 ♂ 10 ♀	24.4 ± 2.5	171.9 ± 10.2	67.1 ± 10.5	20.0 ± 3.5	2–3:	n = 5	< 6:	n = 10
						3–5:	n = 2	6–12:	n = 3
						> 5:	n = 8	12–24:	n = 2

### Experimental setup

**Fig. 1:**
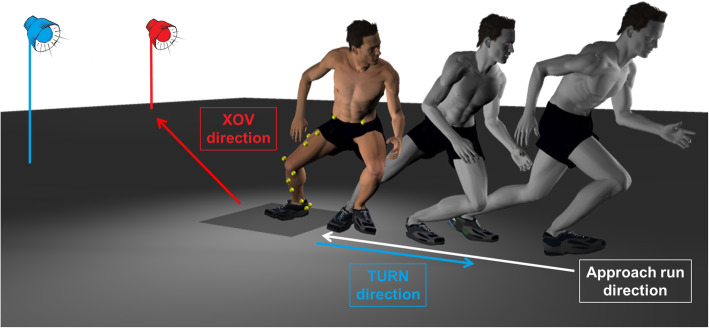
Experimental Setup: Depending on the respective light signal (illustrated in red and blue for the sake of clarity), participants had to perform a 180° turning movement (TURN, depicted here) or a 25° crossover-cutting movement (XOV) on a force platform after a straight approach run

Participants started each trial with a straight approach run with a velocity of 3.5 ± 0.3 m/s, which was determined via photoelectric sensors (Timer S3, Alge Timing, Palling, Germany) over a distance of two metres at the end of the approach run. When they reached a force plate (OR6-7-2000, AMTI, Watertown, USA), they had to perform one of two different movement tasks with a change of direction: a 180° turning movement (TURN) or a 25° crossover-cutting movement (XOV) (Fig. 1). Which movement had to be performed was indicated by light signals that appeared during the approach run when the participants crossed a photoelectric barrier. The signal appeared approximately 1000 ms before foot strike with the test leg on the force plate, which allowed enough time to perform the desired movements safely, but still presented a challenging task [36]. Before the beginning of the measurement, all subjects performed a number of familiarization trials in order to get used to the velocity of the approach run and to make sure that participants felt safe in the execution of the required tasks. For data collection, the order of the two movements was randomized and participants had to perform 10 trials in each direction. This procedure was repeated in three series of movements in a randomized order: without external stabilization, with a soft brace and with a semi-rigid brace, which were chosen as representatives of the respective type of brace. The soft brace (MalleoTrain® S open heel, Bauerfeind AG, Zeulenroda, Germany) consisted of an elastic knitted sleeve and an adjustable elastic strap, while the semi-rigid brace (MalleoLoc®, Bauerfeind AG, Zeulenroda, Germany) was composed of a plastic splint that was attached to the medial and lateral side of the ankle joint with two hook-and-loop straps.

Only TURNs were used for further analysis, because no significant inversion loading was expected in XOVs. XOVs were only included to impede early anticipation of the upcoming task in order to simulate more game-like movements [36].

### Data acquisition and analysis

A 3D motion analysis system with 12 cameras (Vicon Motion Systems, Oxford, UK) was used to track and record marker movement with a sampling frequency of 200 Hz. Reflective skin markers with a diameter of 14 mm were attached to the anterior and posterior superior iliac spines, the lateral and medial knee joint space, and the lateral and medial malleolus. In addition, clusters of three markers were attached to the thigh, shank and calcaneus. The placement of markers directly onto the skin over calcaneus enabled us to determine ankle joint angles as opposed to the movement of the shoe relative to the shank. For this purpose, three holes were cut into the shoes (Adidas Spezial, ADIDAS AG, Herzogenaurach, Germany) on the medial, lateral and posterior side of the heel cap [36–38]. Markers on the medial and lateral malleolus were only attached to the skin during static trials before the beginning of the measurements. Their position was recorded and saved relative to the position of the shank cluster in VICON Nexus 1.7 (Vicon Motion Systems, Oxford, UK). In all dynamic trials, the position of the malleolus markers was reconstructed from the shank markers, because direct placement on the skin was not possible when participants wore a brace. Three-dimensional movement of the ankle joint complex was determined following ISB recommendations [39].

During foot contact with the ground on the force plate, kinetic data were recorded with 2000 Hz and synchronized with the motion capture data. Kinematic and kinetic data were filtered with a 4th order, zero-lag Butterworth low-pass filter with a cutoff frequency of 15 Hz. Three-dimensional external joint moments were calculated with an inverse dynamic approach. Ankle inversion angles at foot strike as well as maximum ankle inversion angles, velocities and moments during the first 200 ms of ground contact were determined using custom-made scripts in Matlab (Version R2015b, The MathWorks, Natick, USA) using BTK toolbox [40]. The time interval of 200 ms after foot strike was chosen as this is the period in which actual ankle sprains are likely to occur [41, 42].

Electromyographic (EMG) data from four lower limb muscles – m. tibialis anterior, m. soleus, m. gastrocnemius lateralis and m. peroneus longus – were recorded with a wireless EMG-system (myon RFTD-E08; myonAG, Baar, Switzerland) at 2000 Hz. The skin was shaved, abraded and cleaned with alcohol to reduce skin impedance. Bipolar pre-gelled Ag/AgCl electrodes with a sensor area of 10 mm² (BlueSensor P, Ambu, Ballerup, Denmark) were then attached to the skin according to SENIAM guidelines [43]. A constant time delay of 15 ms between kinematic and neuromuscular data due to the wireless signal transmission of the EMG signals was corrected after data acquisition. After visual inspection, EMG data were filtered using a 4th order, zero-lag Butterworth band-pass filter (10–750 Hz). Neuromuscular activity in the preparatory phase of a movement as well as during early stance have been reported to be crucial for successful ankle joint control [24]. Therefore, the activity of muscles surrounding the ankle joint was analysed from 100 ms before to 200 ms after foot strike [44, 45]. For a more detailed analysis, this time frame was subdivided into three time intervals with a length of 100 ms: (1) a 100 ms interval before foot strike, (2) the first 100 ms after foot strike, (3) from 100 ms after foot strike to 200 ms after foot strike. Root mean square (RMS) values were determined separately for each time interval.

RMS values were then normalized to straight running. For this purpose, all participants were tested under constant running conditions without wearing an ankle brace before the beginning of the measurement. During running with constant speed (3.5 m/s), RMS values of all muscles were determined for a minimum of three entire stride cycles after applying the same filtering routine as described above. The mean values of these running cycles were used for muscle-specific EMG normalization during TURNs.

For statistical analysis, two-way repeated measures analyses of variance (ANOVAs) were calculated with the within-subject factor CONDITION (no brace, soft brace, semi-rigid brace) and the between-subject factor GROUP (control, FI, FMI). Post-hoc t-tests were calculated where the ANOVAs indicated significant influences. Dependent variables were ankle inversion angles at foot strike, maximum ankle inversion angles, velocities and moments during the first 200 ms of ground contact and RMS values of the four muscles in the three time intervals described above. Alpha-levels were Bonferroni corrected to 0.003 for the ANOVAs and 0.017 for post-hoc comparisons. In addition to tests for statistical significance, effect sizes were calculated. Eta-square values (small effect: 0.01 < η2 < 0.06, medium effect: 0.06 < η2 < 0.14, large effect: η2 > 0.14) [46] were determined for ANOVAs and Cohen’s d values (trivial effect: d < 0.2, small effect: 0.2 < d < 0.5, medium effect: 0.5 < d < 0.8, large effect: d > 0.8) [47] were calculated for post-hoc comparisons.

## Results

### Kinematics and kinetics

The application of braces had a significant main effect on maximum inversion angles (*p* < 0.001, *η²* = 0.371), maximum inversion velocities (*p* = 0.001, *η²* = 0.165) and maximum inversion moments (*p* < 0.001, *η²* = 0.269). Ankle inversion angles at foot strike (*p* = 0.066, *η²* = 0.064) were not significantly influenced by brace application. No significant interaction effects between GROUP and CONDITION were found (*p* ≥ 0.077, *η²* ≤ 0.097).

In the absence of GROUP x CONDITION effects, no post-hoc t-tests to determine the effect of brace application on specific groups were calculated. However, in order to test our second hypothesis, effect sizes for all subgroups were calculated for those parameters which were significantly affected by brace application.

**Fig. 2:**
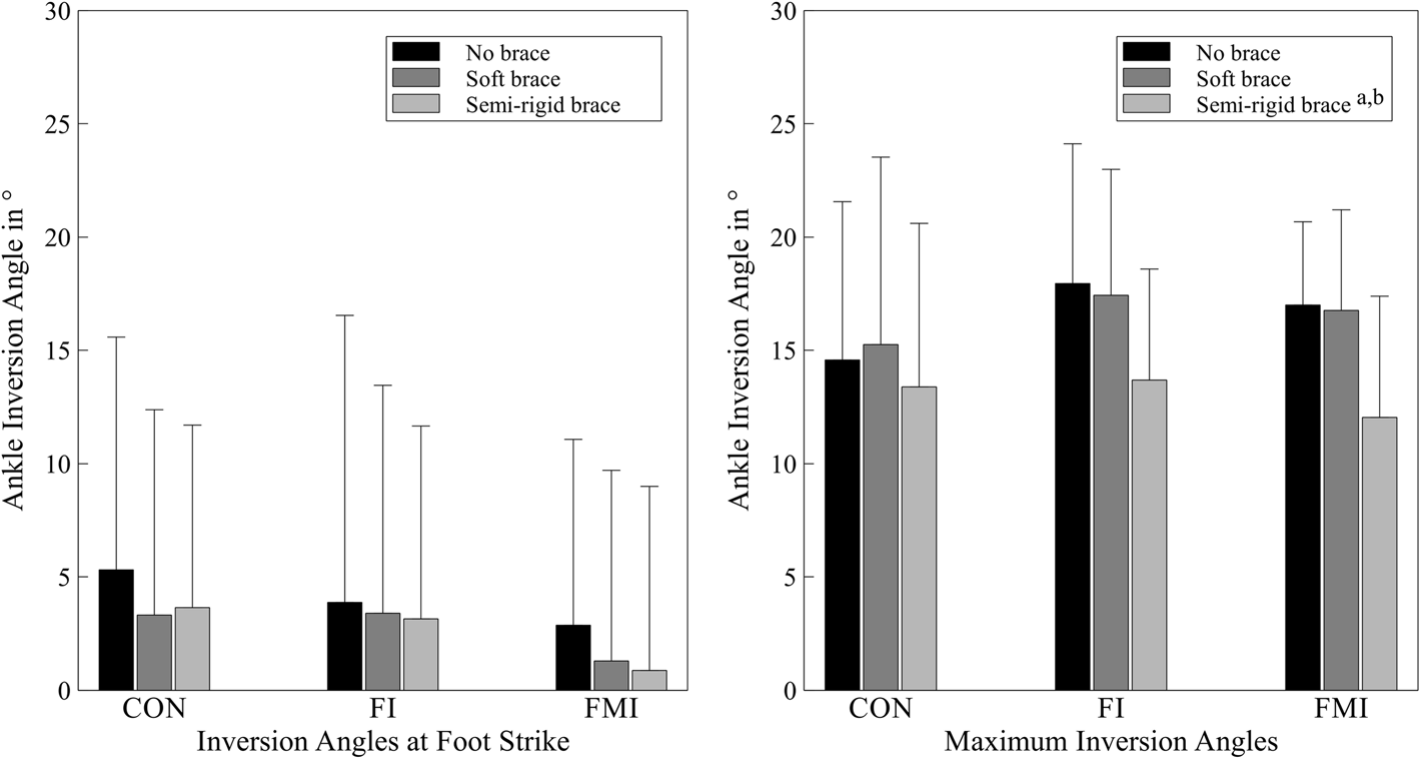
Ankle inversion angles at foot strike (left) and maximum ankle inversion angles (right) of all three groups without a brace, with the soft brace and the semi-rigid brace. ^a^ indicates a significant difference to the condition without a brace, ^b^ indicates a significant difference to the condition with the soft brace

Maximum inversion angles decreased with the application of the semi-rigid brace in comparison to the condition without a brace (*p* < 0.001, *d* = 0.607) and the soft brace (*p* < 0.001, *d* = 0.581). Maximum inversion angles in the FMI-group decreased with large effect sizes (semi-rigid vs. no brace: *d* = 1.080; semi-rigid vs. soft: *d* = 0.958) and in the FI-group with medium effects sizes (semi-rigid vs. no brace: *d* = 0.765; semi-rigid vs. soft: *d* = 0.715), while the control group showed trivial and small effects (semi-rigid vs. no brace: *d* = 0.164; semi-rigid vs. soft: *d* = 0.238) (Fig. 2).

**Fig. 3:**
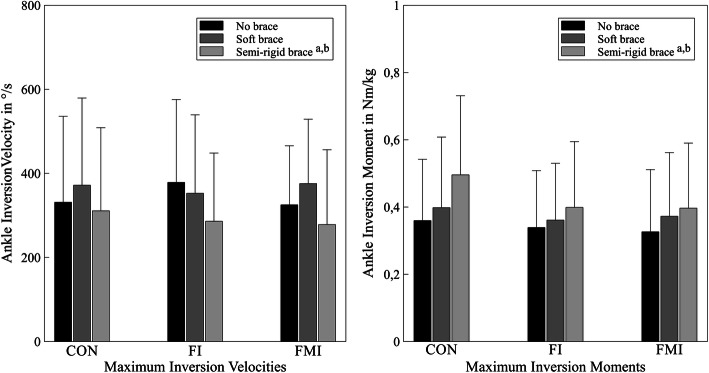
Maximum ankle inversion velocities (left) and moments (right) of all three groups without a brace, with the soft brace and the semi-rigid brace. ^a^ indicates a significant difference to the condition without a brace, ^b^ indicates a significant difference to the condition with the soft brace

Maximum inversion velocities were also reduced when participants wore the semi-rigid brace both in comparison to the condition without a brace (*p* = 0.006, *d* = 0.303) and to the condition with the soft brace (*p* < 0.001, *d* = 0.424). Velocities decreased with small and medium effect sizes in the the FMI-group (semi-rigid vs. no brace: *d* = 0.292; semi-rigid vs. soft: *d* = 0.587) and in the FI-group (semi-rigid vs. no brace: *d* = 0.512; semi-rigid vs. soft: *d* = 0.381), while trivial and small effects were observed in the CON-group (semi-rigid vs. no brace: *d* = 0.101; semi-rigid vs. soft: *d* = 0.300) (Fig. 3).

With the semi-rigid brace, maximum inversion moments increased in comparison to trials without a brace (*p* < 0.001, *d* = 0.455) and to trials with the soft brace (*p* = 0.004, *d* = 0.264). These increases showed medium and small effect sizes in the CON-group (semi-rigid vs. no brace: *d* = 0.647; semi-rigid vs. soft: *d* = 0.438), small effect sizes in the FI-group (semi-rigid vs. no brace: *d* = 0.327; semi-rigid vs. soft: *d* = 0.206) and small and trivial effect sizes in the FMI-group (semi-rigid vs. no brace: *d* = 0.372; semi-rigid vs. soft: *d* = 0.126) (Fig. 3).

### EMG

Bracing had a significant main effect on the activation of peroneus longus in the preparatory phase (*p* < 0.001, *η²* = 0.230) and in the first 100 ms of ground contact (*p* = 0.002, *η²* = 0.140). No significant changes were observed in the second 100 ms of ground contact (*p* = 0.440, *η²* = 0.019) or in any of the other muscles (*p* ≥ 0.009, *η²* ≤ 0.110) (Table 2). GROUP x CONDITION effects did not appear (*p* ≥ 0.017, *η²* ≤ 0.145).

**Fig. 4:**
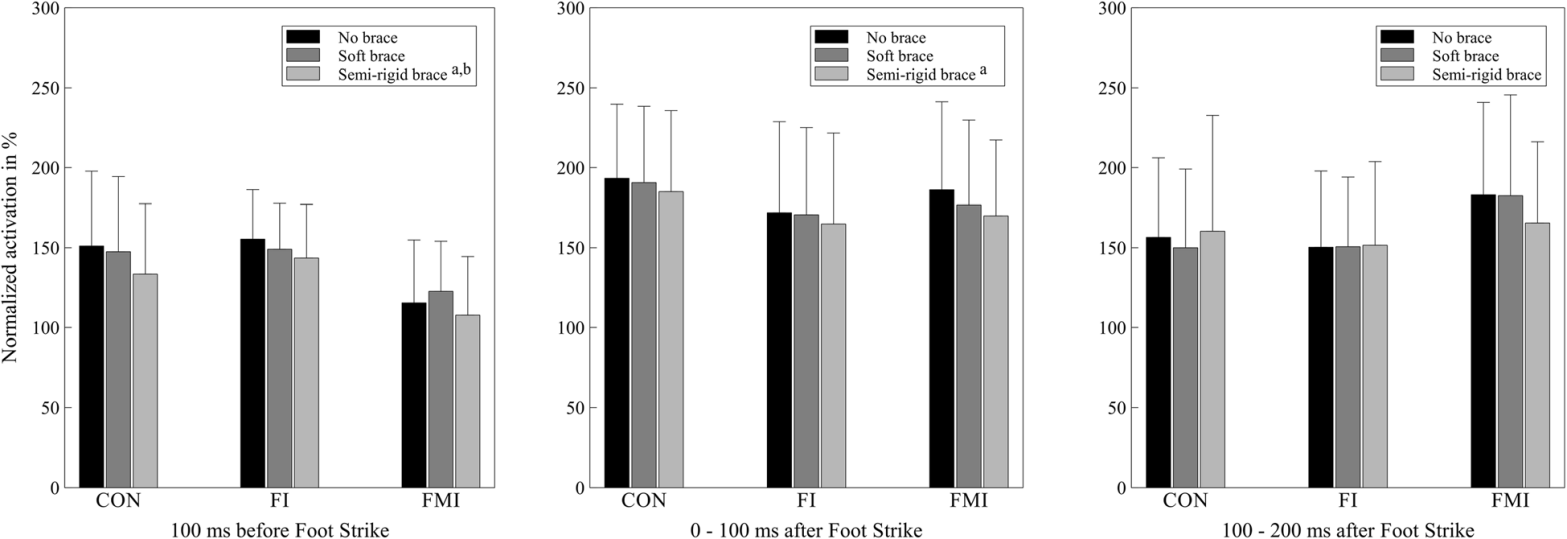
Activation of m. peroneus longus in the 100 ms interval before foot strike (left), the first 100 ms after foot strike (middle) and from 100 to 200 ms after foot strike (right) of all three groups without a brace, with the soft brace and the semi-rigid brace. ^a^ indicates a significant difference to the condition without a brace, ^b^ indicates a significant difference to the condition with the soft brace

Preactivation of peroneus longus was decreased when participants wore the semi-rigid brace compared to trials with no brace (*p* < 0.001, *d* = 0.296) and the soft brace (*p* < 0.001, *d* = 0.293). These decreases reached small effect sizes in the CON-group (semi-rigid vs. no brace: *d* = 0.389; semi-rigid vs. soft: *d* = 0.309) and the FMI group (semi-rigid vs. no brace: *d* = 0.202; semi-rigid vs. soft: *d* = 0.436) and small and trivial effect sizes in the FI-group (semi-rigid vs. no brace: *d* = 0.368; semi-rigid vs. soft: *d* = 0.176) (Fig. 4; Table 2).

In the first 100 ms of ground contact, activation of peroneus longus was reduced during trials with the semi-rigid brace in comparison to trials without external support (*p* = 0.001, *d* = 0.204). Values in the FMI-group were reduced with a small effect size (*d* = 0.316), while the CON-group (*d* = 0.172) and the FI-group (*d* = 0.124) displayed trivial effect sizes (Fig. 4; Table 2).

**Table 2:** Muscular activation levels (Mean ± SD)

	**Control group**			**FI-group**			**FMI-group**		
	**Without**	**Soft**	**Semi-rigid**	**Without**	**Soft**	**Semi-rigid**	**Without**	**Soft**	**Semi-rigid**
M. tibialis anterior (Mean ± SD), % stride cycle									
Preactivation	92.5 ± 45.8	88.5 ± 43.1	84.9 ± 42.0	83.0 ± 30.9	75.0 ± 30.9	83.6 ± 33.5	93.9 ± 33.6	88.5 ± 30.5	87.7 ± 33.0
0–100 ms after foot strike	92.7 ± 70.8	95.8 ± 68.8	89.1 ± 60.3	90.7 ± 35.9	85.0 ± 35.7	91.9 ± 43.2	85.3 ± 30.0	85.7 ± 23.3	89.8 ± 29.1
100–200 ms after foot strike	84.5 ± 61.8	90.3 ± 56.4	83.0 ± 52.5	79.1 ± 35.7	72.3 ± 38.0	75.5 ± 41.3	65.6 ± 28.3	68.2 ± 21.6	65.3 ± 21.9
M. peroneus longus (Mean ± SD), % stride cycle									
Preactivation ^a,b^	151.1 ± 46.7	147.5 ± 46.9	133.4 ± 44.0	155.4 ± 30.8	149.0 ± 28.7	143.6 ± 33.5	115.4 ± 39.4	122.6 ± 31.4	107.7 ± 36.8
0–100 ms after foot strike ^a^	193.3 ± 46.4	190.6 ± 47.8	184.9 ± 50.8	171.9 ± 56.9	170.6 ± 54.5	164.8 ± 56.8	186.1 ± 55.1	176.7 ± 53.1	169.9 ± 47.4
100–200 ms after foot strike	156.5 ± 49.7	150.0 ± 49.1	160.3 ± 72.4	150.3 ± 47.6	150.6 ± 43.5	151.6 ± 52.2	183.0 ± 57.9	182.4 ± 63.2	165.5 ± 50.7
M. soleus (Mean ± SD), % stride cycle									
Preactivation	104.3 ± 31.5	104.4 ± 38.5	100.5 ± 30.7	115.4 ± 40.4	107.6 ± 39.5	97.6 ± 24.6	82.5 ± 34.2	93.5 ± 37.4	88.5 ± 31.4
0–100 ms after foot strike	136.6 ± 26.8	131.1 ± 25.3	131.0 ± 21.9	137.6 ± 18.2	145.2 ± 36.4	144.2 ± 34.8	127.9 ± 33.2	130.5 ± 27.3	115.0 ± 22.7
100–200 ms after foot strike	146.1 ± 33.0	142.6 ± 34.2	130.9 ± 33.5	163.4 ± 51.8	165.5 ± 47.4	164.0 ± 51.3	176.5 ± 48.8	156.7 ± 45.5	152.1 ± 42.9
M. gastrocnemius lat. (Mean ± SD), % stride cycle									
Preactivation	143.3 ± 49.7	147.9 ± 54.0	137.3 ± 55.5	146.9 ± 42.4	146.5 ± 48.0	142.7 ± 49.8	141.1 ± 45.9	135.7 ± 39.9	131.3 ± 39.0
0–100 ms after foot strike	107.8 ± 24.5	108.6 ± 27.9	101.7 ± 25.5	103.6 ± 39.8	115.6 ± 47.6	110.6 ± 40.6	106.1 ± 43.2	108.0 ± 41.3	102.8 ± 32.3
100–200 ms after foot strike	126.4 ± 39.2	118.3 ± 40.6	110.4 ± 34.9	132.9 ± 54.6	133.9 ± 58.7	135.5 ± 62.0	153.6 ± 58.8	153.1 ± 72.0	130.4 ± 41.8

^a^ indicates a significant difference between the condition with the semi-rigid brace and the condition without a brace

^b^ indicates a significant difference between the condition with the semi-rigid brace and the condition with the soft brace

## Discussion

As expected, ankle bracing had significant effects on joint kinematics and kinetics as well as on peroneal activation before and after ground contact. Specifically, the application of the semi-rigid brace led to reductions in maximum ankle inversion angles and velocities during injury-relevant turning movements, which are very challenging for dynamic stabilization of the ankle joint complex. In addition, participants decreased their peroneal activation when wearing the semi-rigid brace. Interestingly, the effects of the semi-rigid brace were visible not only in comparison to the condition without external ankle support but also compared to the condition in which participants wore a soft brace. Thus, particularly the mechanical rigidity of a brace seems to play an essential role in limiting ankle joint angles and velocities and consequently in injury prevention. The observed reduction in peroneal activation is likely to be a consequence of the protective effect of the brace on the ankle joint.

Significant brace by group interactions did not appear. However, particularly in regard to ankle joint kinematics, the magnitudes of the effects of ankle bracing differed between the three study groups, which is why we will include this aspect in the following discussion.

### Effects of ankle bracing on ankle joint kinematics

The abovementioned results are in agreement with previous studies, which have also shown a reduction of inversion angles and velocities with the application of ankle braces in a variety of different movement tasks: Both a lace-up brace and a semi-rigid brace successfully restricted maximum ankle inversion angles during walking and running on a laterally tilted treadmill [48]. During simulated inversion movements on tilt platforms, decreased maximum inversion angles and velocities have been observed with soft, semi-rigid and rigid braces including lace-up and hinged braces in people with and without CAI [16, 17, 20]. In a recent study, however, female basketball players showed reduced maximum inversion angles in basketball-specific cutting movements only with a hinged brace, while a lace-up brace did not provoke the same changes [49]. In accordance with these findings, the importance of the rigidity of an ankle brace for stabilization of the ankle joint complex in highly dynamic, injury-relevant movements is highlighted by the results of the present study. Only the semi-rigid brace effectively reduced ankle joint angles and velocities, while the soft brace, which consisted of an elastic knitted sleeve and adjustable straps, had no significant effects on ankle joint kinematics.

Interestingly, in the present study, brace application had larger effects on participants with unstable ankle joints than on participants with healthy ankle joints. Maximum ankle inversion angles were decreased with large effects in the FMI-group and with medium effects in the FI-group with the semi-rigid brace, while decreases in the CON-group were trivial. Maximum ankle inversion velocities were reduced with the semi-rigid brace in the FI-group with medium effect sizes, in the FMI group with small effect sizes and with only trivial effects in the CON-group. These are important findings considering that people with unstable ankle joints have displayed increased inversion angles during walking, running and jump landing and that this has been proposed to be a reason for repeated sprains in this population [50–53]. Similarly, although not statistically significant, maximum inversion angles during TURNs in the present study were considerably higher in both the FI- (17.9°) and the FMI-group (17.0°) compared to controls (14.6°) when participants wore no external ankle support. A reduction of these increased values may be an important factor in the prevention of recurrent sprains. Indeed, with the semi-rigid brace, inversion angles were reduced substantially in both the FI- and FMI-group, while values in the CON-group hardly changed, resulting in very similar values in all study groups (CON: 13.4°, FI: 13.7°, FMI: 12.1°). Thus, even in a challenging change of direction task, the application of a semi-rigid brace led to a reduction of ankle inversion angles in people with CAI to the level of healthy controls.

### Effects of ankle bracing on different subgroups of people with CAI

We expected that participants with a combination of FI and MI might display higher ankle inversion angles and velocities without external support than participants with isolated FI. This assumption was based on results from a previous study, which showed that particularly the presence of mechanical instability was related to increased maximum inversion angles during artificially induced ankle inversions on a tilt platform [33]. As a consequence, we hypothesized that those with FI and MI would benefit more strongly from the semi-rigid brace due to its design, which should be most suitable to counteract the mechanical insufficiencies inherent in this group. However, no systematic differences appeared between participants in the FI- and FMI-group in the present study in the condition without a brace. Furthermore, contrary to our hypothesis, brace application had very similar effects on both subgroups of CAI, regardless of their mechanical ankle stability status. Nevertheless, Cohen’s d values of the reductions in maximum inversion angles with the semi-rigid brace were larger for participants with FMI than for participants with FI. This may indicate that people with mechanical insufficiencies may benefit even more from more rigid braces than people with purely functional impairments, although the results of the present study can only document a general protective effect of a semi-rigid brace on both subgroups of people with CAI.

### Relevance for injury prevention

We observed a general protective effect of ankle braces with larger reductions in inversion angles and velocities in participants with CAI than in healthy controls. This may explain why braces seem to reduce ankle sprains mainly in people with previous ankle injuries. Two studies which differentiated between the reduction of ankle sprains in people with and without a previous history of ankle injuries showed that injuries were reduced by more than 50 % exclusively in people with a history of ankle sprains. Injury incidences in people without previous ankle injuries did not change with brace application [54, 55]. It has to be mentioned, however, that two more recent publications reported significant reductions in overall ankle injury rates also in adolescents without previous ankle injuries, which challenges this hypothesis [56, 57]. Similarly, a recent review and meta-analysis indicates that braces may also be effective in primary prevention of ankle injuries in general [9]. Still, the results of the present study suggest that healthy people do not seem to be as reliant on external ankle support as people with unstable ankle joints in movements with a change of direction.

### Effects of ankle bracing on neuromuscular activation

In contrast to the observed kinematic changes, the effects of ankle braces on neuromuscular activation do not seem to support the prevention of inversion injuries at first sight. Application of the semi-rigid brace lead to significant reductions of peroneal activation levels before foot strike and in the first 100 ms after foot strike. The soft brace did not provoke any significant changes in neuromuscular activation and no other muscle showed different activation levels with either of the tested ankle braces (see Table 2). Restriction of frontal plane movement is the primary purpose of ankle brace application [58] and ankle braces therefore provide the highest amount of mechanical stiffness in this direction. Reduced peroneal activation levels suggest that the neuromuscular system responded specifically to the stabilizing effect of the semi-rigid ankle brace in the frontal plane and demonstrate that participants reacted to the additional passive resistance of the brace with a decrease of active joint stabilization [13].

Decreases in peroneal activity with the application of ankle braces have also been shown previously in walking, jogging, running and during functional exercises [27–29]. Furthermore, reduced peroneal activity was found in simulated inversion sprains on a tilt platform during walking with a semi-rigid ankle brace [16]. These effects appeared consistently in people with and without CAI. Similarly, as illustrated in Fig. 4, the present study indicates a general trend for decreased peroneal activation levels from the condition without a brace over the condition with the soft brace to the trials with the semi-rigid brace. The amount of mechanical resistance of the brace seems to have a direct influence on the reductions in peroneal activation levels [13].

Overall, while other braces or taping may affect ankle joint biomechanics via different pathways, the results of the present study suggest that the observed changes in movement kinematics and neuromuscular activation were primarily due to the mechanical properties of the semi-rigid brace. Braces have also been proposed to stimulate skin mechanoreceptors and consequently lead to improved sensorimotor control of the ankle joint in people with recurrent sprains [55]. However, if the effect of an ankle brace was mainly based on the stimulation of skin receptors, one would expect that at least the soft brace, which encloses the ankle joint more tightly than the semi-rigid brace, would stimulate neuromuscular activation of muscles surrounding the ankle joint and would also have an effect on ankle joint kinematics [22]. The fact that only the semi-rigid brace yielded significant effects as well as the finding that active joint protection was even decreased with the application of ankle braces support the hypothesis that it is the mechanical stiffness provided by the brace that plays the central role in injury prevention [11].

For a complete representation of the findings of the present study, the following points need to be addressed. First, while we can undoubtedly report different effects of the two braces used in this study, we have to be careful not to draw generalized conclusions about the effects of soft and semi-rigid braces. However, the observed differences between trials with MalleoTrain® S open heel and trials with MalleoLoc® may be seen as an indication that braces which belong to the same categories may affect movements with a change of direction in similar ways. Second, in spite of decreased inversion angles and velocities, ankle inversion moments increased significantly when participants wore the semi-rigid brace. Unfortunately, the explanation for these increased moments remains unclear. Neither differences in angular acceleration of the ankle joint nor changes in ground reaction forces and foot placement could provide a conclusive explanation for the increased moments in additionally performed analyses. Third, while we report a tendency for increased maximum ankle inversion angles in people with ankle instability in the condition without a brace in the present study, an earlier project, in which we used identical inclusion criteria and a very similar experimental setup, provided different results [59]. People with unstable ankles showed smaller maximum ankle inversion angles than healthy controls in movements with a change of direction, which was considered to be a safety mechanism under conditions that were considered as potentially harmful for the ankle joint complex. While maximum inversion angle values in participants with ankle instability are very similar in the two studies, values in the control group seem to be responsible for the discrepancies. It is possible that minor methodological differences may have accounted for different outcomes. While the earlier study included three different movement types and the velocity of the approach run was set at 4 ± 0.3 m/s, participants in the current study only faced two different movements with a change of direction after a slightly slower approach run, which may have presented a slightly easier task. While these different findings emphasize the general need for detailed descriptions of experimental and methodological approaches [60, 61], the main result of the present study, a reduction in inversion angles and velocities with the application of a semi-rigid brace, remains unaffected.

## Conclusions

The present study has important implications for clinicians, coaches and people with CAI. Our data reveal that maximum ankle inversion angles and velocities can be reduced during injury-relevant movements with a change of direction by the application of a semi-rigid brace. An elastic soft brace, however, did not significantly influence ankle joint kinematics in highly dynamic run-and-cut movements, which most likely was due to its lower mechanical stiffness. We therefore strongly recommend the use of a semi-rigid ankle brace for the prevention of recurrent sprains.

## Data Availability

The datasets used and/or analysed during the current study are available from the corresponding author on reasonable request.

## Abbreviations

ANOVA: Analysis of variance
CAI: Chronic ankle instability
CAIT: Cumberland Ankle Instability Tool
CON: Control
EMG: Electromyography/Electromyographic
FI: Functional ankle instability
FMI: Functional and mechanical ankle instability
MI: Mechanical ankle instability
RMS: Root mean square
TURN: 180° turning movement
XOV: 25° crossover-cutting movement
